# Cost-utility of Intravenous Immunoglobulin (IVIG) compared with corticosteroids for the treatment of Chronic Inflammatory Demyelinating Polyneuropathy (CIDP) in Canada

**DOI:** 10.1186/1478-7547-8-14

**Published:** 2010-06-17

**Authors:** Gord Blackhouse, Kathryn Gaebel, Feng Xie, Kaitryn Campbell, Nazila Assasi, Jean-Eric Tarride, Daria O'Reilly, Colin Chalk, Mitchell Levine, Ron Goeree

**Affiliations:** 1 PATH Research Institute, McMaster University, Hamilton, Ontario, Canada; 2 Department of Clinical Epidemiology & Biostatistics, McMaster University, Hamilton, Ontario, Canada; 3 Centre for Evaluation of Medicines, St. Joseph's Healthcare, Hamilton, Ontario, Canada; 4 Department of Neurology and Neurosurgery, McGill University, Montréal, Québec, Canada

## Abstract

**Objectives:**

Intravenous immunoglobulin (IVIG) has demonstrated improvement in chronic inflammatory demyelinating polyneuropathy (CIDP) patients in placebo controlled trials. However, IVIG is also much more expensive than alternative treatments such as corticosteroids. The objective of the paper is to evaluate, from a Canadian perspective, the cost-effectiveness of IVIG compared to corticosteroid treatment of CIDP.

**Methods:**

A markov model was used to evaluate the costs and QALYs for IVIG and corticosteroids over 5 years of treatment for CIDP. Patients initially responding to IVIG could remain a responder or relapse every 12 week model cycle. Non-responding IVIG patients were assumed to be switched to corticosteroids. Patients on corticosteroids were at risk of a number of adverse events (fracture, diabetes, glaucoma, cataract, serious infection) in each cycle.

**Results:**

Over the 5 year time horizon, the model estimated the incremental costs and QALYs of IVIG treatment compared to corticosteroid treatment to be $124,065 and 0.177 respectively. The incremental cost per QALY gained of IVIG was estimated to be $687,287. The cost per QALY of IVIG was sensitive to the assumptions regarding frequency and dosing of maintenance IVIG.

**Conclusions:**

Based on common willingness to pay thresholds, IVIG would not be perceived as a cost effective treatment for CIDP.

## Introduction

Chronic inflammatory demyelinating polyneuropathy (CIDP) is an acquired immune-mediated inflammatory disorder that targets the myelin sheaths that wrap the nerves of the peripheral nervous system. The motor weakness symptoms of CIDP resemble those of Guillain-Barre syndrome (GBS), and CIDP is sometimes considered to be a chronic counterpart of GBS[1]. The course of CIDP may be chronic progressive, stepwise, or monophasic. CIDP can occur at all ages and in both sexes, but is more common in older individuals and males. It is believed that the older age group is more likely to have a chronic progressive course of CIDP, and in younger patients, a relapse remitting course[2]. The prevalence rate of CIDP has been reported to be between 1.0 to 1.9 per 100,000 population[3,4].

CIDP has both motor and sensory symptoms, with motor being the predominant feature. There is symmetrical involvement of both arms and legs, including both proximal and distal muscles, resulting in global muscle weakness and a general reduction or absence of deep tendon reflexes[2]. Occasionally, muscle weakness becomes profound, and patients are unable to walk[5]. A prevalence study conducted by Lunn and colleagues[3] reported that 54% of patients had been severely disabled at some point in the past, and 13% were still severely disabled at the time of the prevalence assessment.

Patients with CIDP show improvement after treatment with corticosteroids and Plasma Exchange (PE),[6,7] but both treatments have disadvantages. Due to the chronic nature of the disease, long-term use of corticosteroids is usually required, and this carries the risk of numerous Adverse Events (AEs) and serious adverse events (SAEs)[8]. The benefit from PE is usually transient, therefore it is usually employed concomitantly with other therapy[7]. Also, PE must be carried out in specialized centres, and the repeated procedures require good vascular access[9].

In September 2008, the Food and Drug Adminsitration (FDA) granted Talecris Biotherapeutics supplemental licenses for their IVIG products to include CIDP as an indication[10]. The Health Products and Food Branch of Health Canada granted their approval for this indication in October 2008[11]. IVIG has demonstrated improvement in CIDP patients in placebo-controlled trials[9,12-14]. However, IVIG is also expensive. A recent report estimated the annual IVIG maintenance costs to be over $70,000 in Canada[15].

Canada has one of the highest per capita rates of consumption of IVIG in the world, and the consumption rate has been increasing annually over the past decade[16,17]. Escalating cost, increasing demand for an expanding number of indications, and a recent IVIG shortage has prompted Canada to adopt new approaches to manage and prioritize IVIG use. Assessing the impact of IVIG in patients with CIDP has been identified as a priority. This is because of its relatively high utilization rates in Canada, the potential availability of alternative treatments, and the uncertainty of a therapeutic advantage over alternative therapy.

The objective of this study is to evaluate the cost-utility of IVIG compared to corticosteroids for the treatment of CIDP in Canada.

## Methods

### Overview

A cost-utility analysis was conducted using a Markov model to compare IVIG to corticosteroids for the treatment of CIDP. The population entering the model are assumed to be 54 years of age and weighing 75 kg. These assumptions are based on the average age and weight of patients in the trial that compared IVIG and corticosteroid treatment in patients with CIDP[18]. The analysis is taken from the perspective of a Canadian publicly funded health care system. Although IVIG forms part of the budget for Canadian Blood Services (CBS), its costs are borne by Canadian public health care payers as part of their payments to CBS[19]. The effectiveness measure is quality adjusted life years (QALY). In the basecase analysis, the time horizon of the model is set to five years. Alternate time horizons are assumed in sensitivity analyses. Both costs and effects were discounted at a rate of 5% annually.

### Model structure

**Figure 1:**
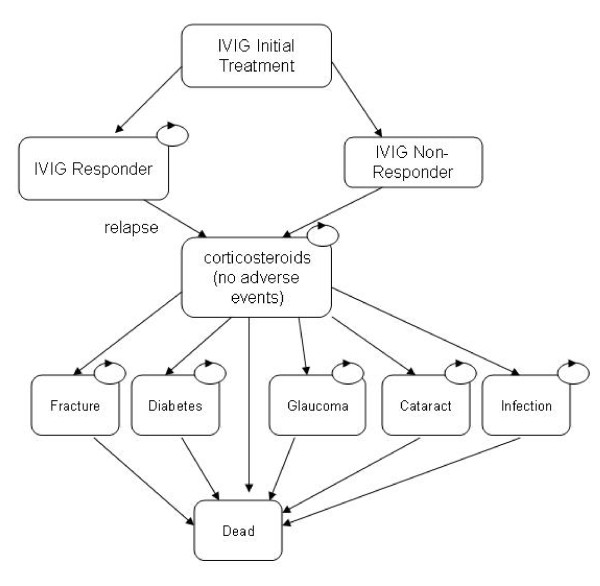
**Structure of IVIG treatment arm of the model**.

**Figure 2:**
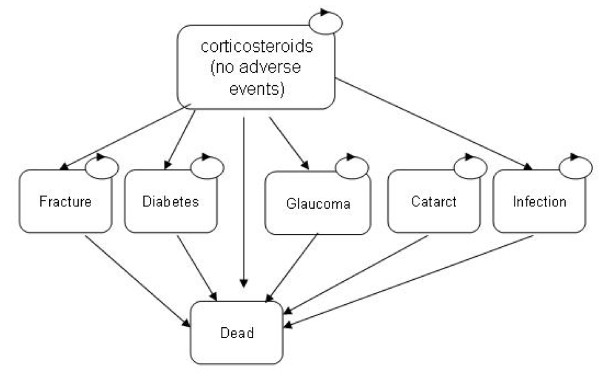
**Structure of corticosteroid treatment arm of the model**.

The structure of the model, including the transitions between health states, is presented in Figures 1 and 2. Figure 1 presents the model structure for the IVIG treatment strategy. Each box represents different health states in the model. Transitions between one health state to another are indicated by straight arrows in the figures. Circled arrows indicate patients can remain in a health state from one model cycle to the next. As shown, all patients enter the model in the IVIG initial treatment health state. Each model cycle represents 12 weeks of time. After this initial twelve week cycle, a proportion of patients are either IVIG responders or IVIG non-responders. Patients who respond to treatment are assumed to receive maintenance IVIG each twelve week cycle until they relapse, and therefore no longer respond to treatment. Once patients relapse, they are assumed to switch to corticosteroid treatment. Patients not responding to initial IVIG treatment are also assumed to switch to corticosteroid treatment.

Once patients start corticosteroid treatment, they are at risk of a number of AE's in each twelve week cycle. The AEs used in the model included fracture, diabetes, glaucoma, cataract and serious infection. Though this is not an exhaustive list of side-effects associated with steroid use, we evaluated these as they were incorporated into an economic evaluation of corticosteroids for the treatment of rheumatoid arthritis[20]. This study was used as the source for a number of AE related model inputs. Once patients have an AE, it is assumed that patients discontinue steroid treatment. It is assumed that once treatment is stopped, HbA1C (diabetes), and elevated intraocular pressure (glaucoma) return to normal. Furthermore, it is assumed that these conditions lasted for 1 year duration before discovery and steroid discontinuation. For each adverse event, patients are assigned an increased risk of mortality, increased costs, and a reduction in quality of life.

Figure 2, represents the model structure for the corticosteroid treatment strategy. As shown, it is similar to the structure of the IVIG arm, except no distinction is made between steroid responders and steroid non-responders. There are a number of reasons why no distinction is made. First, the one clinical trial comparing IVIG with corticosteroids in CIDP patients[18] did not report treatment response or relapse as an outcome. Second, because IVIG treatment is so much more expensive than corticosteroids, it is more important to distinguish the proportion of patients that respond and therefore incur maintenance treatment costs, compared to corticosteroids. Finally, the only study that compared utility values in IVIG and corticosteroid treated CIDP patients[21] did not report utility values by responder status.

### Model Inputs

A number of different model input parameters were used to populate the model. These include: initial IVIG response rate; IVIG relapse rates; corticosteroid AE rates; mortality rates; IVIG treatment costs; corticosteroid treatment costs; AE related costs and finally, utility values associated with treatments and AEs. These are discussed below.

#### IVIG response and relapse rates

A literature review was conducted to identify randomized controlled trials that evaluated IVIG for CIDP patients. Six trials were identified that evaluated IVIG and reported response rates[9,12,14,22-25]. Response rates from the IVIG treatment arms of these studies were pooled using a random effects meta-analysis[26]. Table 1 presents details of the meta-analysis. As shown, the pooled IVIG response rate was estimated to be 0.473 (95% CI 0.361, 0.585). The IVIG relapse rate was based upon data from the ICE study[14]. This was the only study that reported relapse rates over a six month period. The 25 week relapse rate for IVIG in this study was estimated to be 13%. This is equivalent to a 12 week relapse rate of 6.5%. The cumulative relapse rate from the 25 week ICE study was extrapolated in the model by applying a constant relapse rate of 6.5% to patients in the IVIG responder health state in each cycle throughout the model time horizon.

**Table 1:** IVIG response rate

Study	n	responders	%	Weight of Study
Zinman (2005)[22]	8	4	50.0%	0.08
Thompson (1996)[12]	7	3	42.9%	0.08
Mendell (2001)[23]	29	11	37.9%	0.20
Hughes (2008)[14]	59	32	54.2%	0.27
Vermuelen (1993)[25]	15	4	26.7%	0.16
Hahn (1996)[9]	30	19	63.3%	0.21
Pooled			47.3% (36.1%, 58.5%)	

#### Corticosteroid adverse event probabilities

The probabilities of corticosteroid related adverse events were taken from a published cost-effectiveness study comparing corticosteroids with Cox-2 inhibitors for the treatment of rheumatoid arthritis[20]. Bae *et al.*[20] used studies by McDougall *et al.*[27] as their source for fracture and cataract probabilities. Saag *et al.*[28] was used as their source for the probabilities of diabetes, glaucoma and serious infection. Table 2 presents the annual corticosteroid AE probabilities used in the model.

**Table 2:** Corticosteroid adverse events

Adverse event	Annual probability
Fracture	0.0098
Diabetes	0.0043
Cataract	0.0114
Glaucoma	0.0008
Serious Infection	0.0035

#### Utilities

Background utility values for the model were based upon utilities from a U.K. general population[29]. Utility values by age and gender are presented in Table 3. Utility gains from IVIG treatment were added to the background utility values, while utility losses from corticosteroid related adverse events were subtracted from background utility values.

**Table 3:** General population utility values

	Utility Values	
Age	Females	Males
35-44	0.91	0.91
45-54	0.85	0.84
55-64	0.81	0.78
65-74	0.78	0.78
75+	0.71	0.75

The incremental gain in utility from IVIG treatment compared to corticosteroid treatment was assumed to be 0.12. This was based on findings from McCrone *et al.*[21] who measured utility at baseline and at 6 weeks in CIDP patients treated with either IVIG or corticosteroids. This utility gain was added to the baseline utility values for all IVIG treated patients for the full duration of the first 12 week model cycle. This utility gain was also applied to patients for the full duration of each subsequent cycle where they remain IVIG responders.

The disutility due to fracture was estimated using an unpublished Canadian model evaluating treatments for corticosteroid induced osteoporosis. This unpublished model is a modification of an osteoporosis model published by Goeree *et al.*[30] The disutility associated with diabetes was estimated using the Ontario Diabetes Economic Model (ODEM)[31]. The ODEM was run for 30 years under different scenarios. First it was run assuming an elevated HbA1C for the first year. Second it was run assuming no elevated HbA1C for the first year. Disutilities were calculated as the difference in utilities predicted by ODEM under these 2 scenarios. Table 4 presents the disutilities associated with fracture and diabetes by first and subsequent years.

**Table 4:** Disutility, incremental mortality, and costs for the first year and subsequent years after fracture and diabetes

	Fracture		Diabetes	
Age	1st year	Subsequent years	1st year	Subsequent years
**Disutility by age group**				
40-44	-0.0833	-0.0293	-0.000179	-0.000333
45-49	-0.0971	-0.0324	-0.000173	-0.000247
50-54	-0.1047	-0.0349	-0.000160	-0.000263
55-59	-0.1068	-0.0371	-0.000074	-0.000681
60-64	-0.1094	-0.0391	-0.000040	-0.000727
65-69	-0.1113	-0.0412	-0.000003	-0.000618
70+	-0.1212	-0.0425	-0.000128	-0.000754
**Incremental mortality by age group**				
40-44	0.0092	0.0001	0.000390	0.000320
45-49	0.0115	0.0001	0.000715	0.000285
50-54	0.0127	0.0001	0.000875	0.000685
55-59	0.0142	0.0002	0.001160	0.000320
60-64	0.0187	0.0003	0.001525	0.000755
65-69	0.0260	0.0006	0.002025	0.001035
70+	0.0541	0.0018	0.002645	0.000850
**Costs by age group**				
40-44	$3,926	$63	$12	$24
45-49	$4,643	$68	$13	$32
50-54	$5,041	$73	$16	$55
55-59	$5,159	$78	$21	$93
60-64	$5,302	$83	$24	$151
65-69	$7,901	$87	$25	$260
70+	$10,880	$744	$27	$341

The disutility associated with the development of cataracts in the model was assumed to be 0.38 while waiting for surgery and 0.10 after surgery[32]. These values were based on a cost-effectiveness study on reducing waiting times for cataract surgery in Ontario[32]. It was assumed that patients would have a 109 day wait for cataract surgery[32]. The disutility for glaucoma was assumed to be 0.061[33]. For serious infection a disutility of 1.0 for two weeks duration was assumed. This assumption was used in Bae *et al.*[20]

#### Mortality

Background mortality rates by age were based on the most recent Canadian life table data[34]. The average of male and female mortality rates were used in the model. The increased risk of death after fracture was derived from the same model which provided the utilities[30]. The increased risk of death from diabetes was estimated using the ODEM[31]. The increased risk of death after fracture and diabetes is presented in Table 4. The acute risk of death from serious infection was based upon data from a Canadian study on in-hospital mortality from community acquired pneumonia[35]. This study reported mortality rates of 0.018 and 0.111 for patients aged between 25-65 and those over 65 respectively. No increase in the probability of death was assumed for the other corticosteroid related adverse events.

#### IVIG costs

The initial and maintenance IVIG treatment cost estimates were based on the dose and frequency of IVIG administration and the cost per each IVIG administration. The dose and frequency of IVIG treatment assumed in the model was based upon the monograph of the product approved for CIDP treatment in Canada[36]. This includes an initial loading dose of 2 grams of IVIG per kg of body weight over two to four days along with maintenance dosing of 1 g/kg over one to two days every three weeks. This is the same dosing regimen used in the study used to estimate IVIG relapse rates[14]. For the purpose of the model, it is assumed that the initial treatment is given as two 1 g/kg doses, and that maintenance IVIG treatment is given as a single 1 g/kg dose every 3 weeks.

The cost per gram of IVIG ($59.19) was provided by Canadian Blood Services (personal communication). The cost per hour for a nurse ($32) was based on the Canadian Salary Survey[37]. Based on a 1 g/kg dose, a 75 kg patient and 3.5 hours of nurse supervision time, the total cost per IVIG administration is calculated as $4551.25. In the initial 12 week cycle patients are assumed to be given two 1 g/kg loading dose treatments of IVIG. They are also assumed to receive 1 g/kg maintenance doses at weeks 3, 6, 9 and 12, resulting in a total cost of $27,307.50 for the initial model cycle. In subsequent twelve week cycles, patients are assumed to have four 1 g/kg IVIG maintenance treatments, resulting in IVIG costs of $18,205. This cost is applied to patients who remain IVIG responders.

#### Corticosteroid Costs

The costs of corticosteroid treatment were based upon the reimbursement rate for a 50 mg pill ($0.0913) and a 5 mg pill ($0.022) of prednisone from the Ontario Drug Benefit formulary[38]. Patients on corticosteroids were assumed to take a bisphosphonate to help protect them from fracture. The cost of etidrocal ($19.99 per 400 mg/500 mg 90 tablet kit) was derived from the Ontario Drug Benefit formulary[38]. Based upon expert opinion, it was assumed that patients would be prescribed 60 mg per day of prednisone for the first 4 weeks of treatment. The dose would then be reduced by 10 mg per day in each of the next 20 weeks. After 24 weeks, the dose was assumed to be tapered down to 5 mg per day. While taking 60 mg of prednisone per day, patients were assumed to take one 50 mg pill and two 5 mg pills per day. While taking 50 mg of prednisone patients were assumed to take a single 50 mg pill. While taking less than 50 mg of prednisone per day, patients were assumed to take multiple 5 mg pills.

An 8%[39] pharmacy markup and a $7.00[39] dispensing fee were incorporated into the corticosteroid costs. Based upon the unit drug costs, pharmacy markup, pharmacy dispensing fee and the assumed treatment regimen, the cost for the first, 2^nd ^and subsequent model cycles are $51.19, $43.57, and $39.87 respectively.

#### Cost of corticosteroid related adverse events

The cost of fracture was estimated using the same model that provided the utility and risk of death after fracture values[30]. The cost of diabetes was estimated using the ODEM[31]. The diabetes and fracture related costs used in the model for the first and subsequent years are provided in Table 4. The cost related to development of cataracts ($6,218) was taken from Hopkins *et al.*[32] and was primarily comprised of surgery costs. The costs related to development of glaucoma ($152) and serious infection ($24,334) were based on the estimates used by Bae *et al.,*[20] inflated to 2008 Canadian dollars. This conversion from U.S. to Canadian dollars was done using the December 2008 currency exchange rate[40]. Inflation from 1999 Canadian dollars to 2008 Canadian dollars was done using the health care component of the consumer price of the consumer price index[41].

### Uncertainty

The variability of cost-effectiveness results according to patient characteristics was assessed using one-way sensitivity analysis. The model was run assuming different patient weights and starting ages. Because patient weight affects IVIG dosing, it also affects the costs. The structural uncertainty of the model was evaluated using one-way sensitivity analyses varying the discount rates and the model duration. The model was also evaluated using different assumptions about the utility gain from IVIG, the extrapolation of IVIG relapse rates, treatment switching for corticosteroid patients after suffering an AE, and on the dosing and frequency of maintenance IVIG treatment.

Parameter uncertainty was evaluated using probabilistic sensitivity analysis and expressed as cost-effectiveness acceptability curves (CEACs) based upon 1000 2^nd ^order Monte Carlo simulations. Beta distributions were used for parameters whose values are constrained between zero and one. These include probability parameters, baseline utility variables and utility weight parameters. Gamma distributions were used for corticosteroid adverse event cost parameters as the values of the cost parameters need to be non-negative. No distributions were applied to the unit costs of IVIG or corticosteroids. Normal distributions were applied to the incremtal utility from IVIG response, along with disutility from corticosteroid AEs, an incremental mortality from adverse events. Selected distributions and parameters values that were used in the probabilistic sensitivity analysis appear in Table 5.

**Table 5:** Probabilsitic parameters

Variable	Mean Value	Distribution (parameters)	95% C.I. based on parameters
IVIG Response Rate	0.473	beta (α = 35.52,β = 39.61)	(0.361, 0.585)
IVIG relapse rate (25 weeks)	0.13	beta (α = 4.03, β = 26.97)	(0.039, 0.266)
IVIG incremental utility	0.12	normal (μ = 0.12,β = 0.08)	(-0.05, 0.29)
Corticosteroid adverse events annual probailites			
Fracture	0.0098	beta (α = 1.20,β = 120.8)	(0.000,0.033)
Diabetes mellitus	0.0043	beta (α = 0.48,β = 111.52)	(0.000,0.022)
Cataract	0.0114	beta (α = 1.39,β = 120.61)	(0.001,0.036)
Glaucoma	0.0008	beta (α = 0.09,β = 111.91)	(0.000,0.008)
Serious Infection	0.0035	beta (α = 0.39,β = 120.8)	(0.000,0.020)
Glaucoma utility weight	0.96	beta (α = 214.32, β = 13.68)	(0.906,0.967)
Cataract utility weight-before surgery	0.62	beta (α = 62, β = 38)	(0.586,0.767)
Cataract utility weight post-surgery	0.90	beta (α = 90, β = 10)	(0.835,0.951)
Glaucoma utility weight	0.96	beta (α = 214.32, β = 13.68)	(0.906,0.967)

## Results

### Basecase

Table 6 presents the basecase cost-effectiveness results. As shown, the total cost for the IVIG treatment arm over the 5 year duration of the model is estimated to be $124,065. This compares with $2,196 for the corticosteroid treatment arm, resulting in an incremental cost of IVIG compared to corticosteroids of $121,869. Over 5 years, IVIG was estimated to have 3.962 QALYs compared to 3.785 for corticosteroids. The resulting incremental cost-utility ratio of IVIG compared to corticosteroids is $687,287 per QALY gained. Based on these results if society is willing to pay $687,287 or more for a QALY, IVIG would be considered the cost-effective treatment. If societal willingness to pay for a QALY is less than $687,287, corticosteroids would be considered the cost-effective strategy.

**Table 6:** Basecase Results

Treatment	Costs	QALYs	Incremental Costs	Incremental QALYs	ICUR
corticosteroids	$2,196	3.785	ref	ref	
IVIG	$124,065	3.962	$121,869	0.177	$687,287

### Uncertainty

One way sensitivity analyses were conducted on a number of patient characteristics (age and weight) and various model assumptions. The results of sensitivity analysis are presented in Table 7. As shown, the cost per QALY of IVIG compared to corticosteroids for patients weighing 35 kg is $327,665, while the cost per QALY of IVIG for patients weighing 95 kg is $867,090. The incremental cost per QALY for patients with a starting age of 35 years is $686,130, while the cost per QALY for patients with a starting age of 75 years is $683,219 per QALY. Using different discount rates or model time horizons had little impact on the cost-utility estimates. Assuming a larger incremental utility impact of IVIG does impact the results. If a 0.25 utility gain is assumed, the cost per QALY becomes $335,038. If patients in the corticosteroid arm are assumed to switch to IVIG after an adverse event the cost per QALY of IVIG becomes $682,309. If no extrapolation of the IVIG relapse beyond 25 weeks is applied to the model, the cost per QALY of IVIG becomes $672,616. In the basecase analysis, it was assumed that maintenance IVIG was given in 1 mg/kg doses, once every 3 weeks. If it is assumed that maintenance IVIG is 1 mg/kg once every 8 weeks, the cost per QALY of IVIG becomes $288,535. If it is assumed that maintenance IVIG is 0.4 mg/kg once every 8 weeks, the cost per QALY of IVIG becomes $148,518.

**Table 7:** Sensitivity analysis

Parameter varied	Incremental Costs (IVIG-corticosteroids)	Incremental QALYs (IVIG-corticosteroids	$/QALY IVIG vs. corticosteroids
**Patient Weight**			
35 kg	$58,102	0.177	$327,665
45 kg	$74,043	0.177	$417,569
55 kg	$89,985	0.177	$507,474
65 kg	$105,927	0.177	$597,378
75 kg	$121,869	0.177	$687,282
85 kg	$137,810	0.177	$777,186
95 kg	$153,752	0.177	$867,090
**Starting Age**			
35 years old	$122,293	0.178	$686,130
45 years old	$122,077	0.178	$686,759
55 years old	$121,504	0.177	$687,371
65 years old	$119,999	0.176	$683,643
75 years old	$116,182	0.170	$683,219
**Discount rate**			
0%	$126,340	0.185	$682,390
3%	$123,542	0.180	$685,346
**Model time horizon**			
1 year	$59,546	0.078	$764,917
3 years	$102,457	0.146	$702,569
5 years	$121,869	0.177	$687,282
10 years	$139,986	0.209	$670,396
20 years	$143,690	0.218	$658,267
**Incremental IVIG utility gain = 0.25**	$121,869	0.364	$335,038
**Assume corticosteroid patients switch to IVIG after adverse event**	$113,444	0.166	$682,309
**Relapse rate for IVIG not extrapolated beyond 25 weeks**	$173,189	0.257	$672,616
**Maintenance IVIG dose and frequency**			
1.0 mg/kg every 3 weeks	$121,869	0.177	$687,282
1.0 mg/kg every 6 weeks	$65,304	0.177	$368,284
1.0 mg/kg every 8 weeks	$51,163	0.177	$288,535
0.4 mg/kg every 3 weeks	$55,661	0.177	$313,905
0.4 mg/kg every 6 weeks	$32,200	0.177	$181,595
0.4 mg/kg every 8 weeks	$26,335	0.177	$148,518

**Figure 3:**
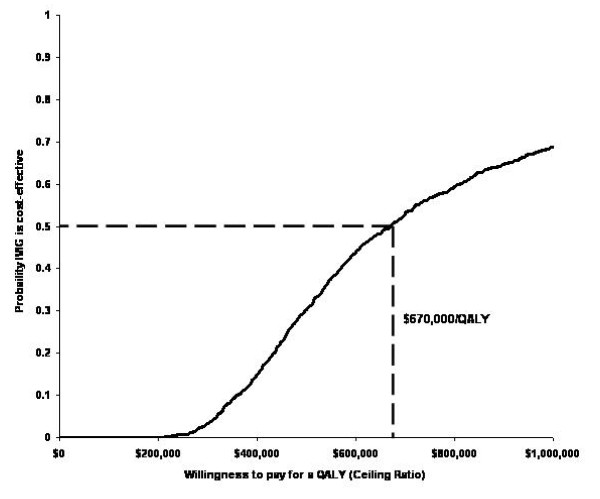
**Cost-effectiveness acceptability curve**.

Figure 3 presents the cost-effectiveness acceptability curve for the IVIG treatment arm using the basecase model assumptions. As shown, at a willingness to pay for QALY threshold of $670,000, the probability that IVIG is cost effective is 50%. At the commonly quoted willingness to pay threshold of $50,000 per QALY, the probability that IVIG is cost-effective is less than 1%.

## Discussion

In this cost-utility analysis in patients with CIDP, the incremental cost of IVIG treatment compared to corticosteroid treatment was estimated to incur $124,065 more costs and result in 0.177 more QALYs compared to the corticosteroid treatment arm over 5 years. The resulting incremental cost-utility ratio of IVIG compared to corticosteroids is $687,287 per QALY gained. The ICUR varied from $327,665 to $867,090 when patient weight was decreased to 35 kg and increased to 95 kg, respectively. Assuming that maintenance treatment with IVIG consists of 0.4 mg/kg doses every 8 weeks instead of 1.0 mg/kg doses every three weeks resulted in a cost per QALY estimate $148,518. Probabilistic sensitivity analysis found that at a willingness to pay for a QALY threshold of $670,000, the probability that IVIG is cost-effective is 50%. Our results are consistent with those from the only other economic evaluation we identified that compared IVIG with corticosteroids for CIDP treatment[21]. In this 6 week trial based economic evaluation, the authors reported that at a willingness to pay threshold of £250,000, there was a 50% chance that IVIG was cost-effective compared to corticosteroids.

The economic analysis has a number of limitations. As is the case for all models, our analysis had to make a number of assumptions. This includes the extrapolation of the non-statistically significant 0.12 (p = 0.07) utility gain from IVIG found by McCrone *et al.*[21] over the five year time horizon of the model. Another limitation is the reliance on this single source of utility gain from IVIG treatment[21]. Another limitation is that the reliance on a single source[20] to define the corticosteroid related adverse events used in the model. Because a public health care payer perspective was taken, indirect costs were not included. If a societal perspective was taken and indirect costs taken into consideration, the cost-utility of IVIG compared to corticosteroids may have been more favourable.

Despite the high costs, IVIG remains a popular treatment in Canada. This is likely due to its potential for better patient outcomes compared to other treatments. Another possible reason is that IVIG funding comes directly from jurisdictional health budgets and do not comprise part of individual hospital budgets.

## Conclusions

IVIG is much more expensive compared to corticosteroids for the treatment of CIDP. Our model estimates the incremental cost per QALY of IVIG compared to corticosteroids to be $687,287. Based on commonly quoted willingness to pay thresholds, IVIG treatment for CIDP is unlikely to be considered a cost-effective use of health care resources. Results varied according to the frequency and dose of IVIG administration.

## Competing interests

CC received funding from Talecris Biotherapeutics Ltd. and is the primary investigator in a multi-centre study funded by Baxter Canada. No payments were received by him or by patients who were enrolled in the study at the time.

## Authors' contributions

GB conceptualized the economic analysis, and was primarily responsible for the data analysis and write up of the study. KG was responsible for conducting the review of the clinical literature review that was used to estimate efficacy variables for the economic analysis. FX helped develop the economic model and assisted with the writing of the manuscript. KC was the information specialist for the manuscript. NA assisted with the overall design of the study and preparation of the manuscript. JET assisted with the preparation of the manuscript. DOR assisted with the preparation of the manuscript. CC provided clinical expertise in the development of the economic model, and assisted with the preparation of the manuscript. ML provided clinical expertise in the development of the economic model and helped write the manuscript. RG assisted with the overall design of the study and preparation of the manuscript. All authors read and approved the final manuscript.
